# Prenatal diagnosis of 7q11.23 microdeletion: Two cases report and literature review

**DOI:** 10.1097/MD.0000000000034852

**Published:** 2023-10-27

**Authors:** Xin Lv, Xiao Yang, Linlin Li, Fagui Yue, Hongguo Zhang, Ruixue Wang

**Affiliations:** a Prenatal Diagnosis Center and Reproductive Medicine Center, The First Hospital of Jilin University, Changchun, China.

**Keywords:** 7q11.23 deletion, chromosomal microarray analysis, prenatal diagnosis, Williams-Beuren syndrome

## Abstract

**Rationale:**

Chromosome microdeletions within 7q11.23 can result in Williams-Beuren syndrome which is a rare autosomal dominant disorder. Williams-Beuren syndrome is usually associated with developmental delay, cardiovascular anomalies, mental retardation, and characteristic facial appearance.

**Patient concerns:**

Two pregnant women underwent amniocentesis for cytogenetic analysis and chromosomal microarray analysis (CMA) because of abnormal ultrasound findings. Case 1 presented subependymal cyst and case 2 presented intrauterine growth restriction, persistent left superior vena cava and pericardial effusion in clinical ultrasound examination.

**Diagnoses:**

Cytogenetic examination showed that the 2 fetuses presented normal karyotypic results. CMA detected 1.536 Mb (case 1) and 1.409 Mb (case 2) microdeletions in the region of 7q11.23 separately.

**Interventions:**

Both couples opted for the termination of pregnancies based upon genetic counseling.

**Outcomes:**

The deleted region in both fetuses overlapped with Williams-Beuren syndrome. To our knowledge, case 1 was the first reported fetus of Williams-Beuren syndrome with subependymal cyst.

**Lessons:**

The genotype-phenotype of Williams-Beuren syndrome is complicated due to the phenotypic diversity. For prenatal cases, clinicians should consider the combination of ultrasonography, traditional cytogenetic, and molecular diagnosis technology when genetic counseling.

## 1. Introduction

Chromosomal microdeletion is usually regarded as an aberrant copy number loss (<5 Mb in size) in a specific subchromosomal region.^[1]^ These microscopic imbalances can hardly be identified by karyotype analysis due to its low resolution but can be detected by molecular cytogenetic technologies, such as chromosomal microarray analysis (CMA). Chromosomal microdeletions are frequently associated with intellectual disability, multiple congenital anomalies, and autism spectrum disorders.^[2–4]^

7q11.23 microdeletion, also known as Williams-Beuren syndrome, generally causes neurodevelopmental disorders.^[5]^ The first cases of Williams-Beuren syndrome were reported in 1960s.^[6,7]^ The prevalence of Williams-Beuren syndrome was approximately 1 in 7500 live births.^[8,9]^ The clinic typical features of Williams-Beuren syndrome are characterized by facial dysmorphisms, supravalvular aortic stenosis, connective tissue abnormalities, abnormalities in multiple endocrine axes, hypercalcemia, and a distinctive neurobehavioral phenotype.^[10,11]^ In addition, individuals with Williams-Beuren syndrome are at risk of sudden cardiac arrest with anesthesia and have higher risk of impaired glucose tolerance.^[12,13]^

Most reports on 7q11.23 microdeletion cases were postnatal cases, while prenatal reports are relatively scarce. The prenatal ultrasound phenotype in 7q11.23 deletions was first described in 2011.^[14]^ Till now, the correlation between prenatal phenotype and 7q11.23 deletions is still not clear. Here, we describe the clinical characterization of 2 cases of 7q11.23 microdeletion prenatally diagnosed using CMA. Meanwhile, we also made a literature review on the prenatal phenotype of pure 7q11.23 microdeletion.

## 2. Materials and Methods

This study was approved by the Ethical Committee of The First Hospital of Jilin University (No. 2021-706) and followed the tenets of the Helsinki Declaration. Informed consent had been obtained from the patient for publication of this case report and accompanying information.

### 2.1. Cytogenetic analysis

Amniotic fluid cells were obtained through amniocentesis after obtaining written informed consent. Amniotic fluid cells were cultured according to standard operating procedure. Chromosome analysis was performed on G-band metaphases at 400 to 500 banding resolution, which were prepared from 10 mL of cultured amniotic fluid cells. Twenty metaphases were analyzed for all samples. The karyotype was described in accordance with the International System for Human Cytogenetic Nomenclature (ISCN 2016).^[15]^

### 2.2. Chromosomal microarray analysis (CMA)

10 mL of uncultured amniotic fluid cells were collected using amniocentesis with informed consent. 5 mL of peripheral blood was collected using a standard vacuum extraction blood-collecting system containing EDTA and heparin for the parents who intended to verify. Genomic DNA was extracted using the QIAamp DNA Mini kit (Qiagen, Hilden, Germany) according to the manufacturer’s protocol. Then the procedures were conducted through CytoScan 750K array (Affymetrix, Santa Clara, CA). The procedure included genomic DNA extraction, digestion and ligation, polymerase chain reaction amplification, polymerase chain reaction product purification, quantification and fragmentation, labeling, array hybridization, washing, and scanning. Thresholds for genome-wide screening were set at ≥200 kb for gains and ≥100 kb for losses. The image data were analyzed using Chromosome Analysis Suite v4.0 software (ThermoFisher Scientific, Waltham, MA). Finally, the detected copy number variations were analyzed according to public databases: CliGen (https://www.clinicalgenome.org/), Database of Genomic Variants (DGV) (http://dgv.tcag.ca/dgv/app/home), DECIPHER (http://decipher.sanger.ac.uk/), online Mendelian inheritance in man (OMIM) (http://www.ncbi.nlm.nih.gov/omim), and International Standards for Cytogenomic Arrays (ISCA) (https://www.iscaconsortium.org/). Genomic positions refer to the Human Genome February 2009 assembly (GRCh37/hg19).

## 3. Case presentation

### 3.1. Case 1

**Figure 1.**
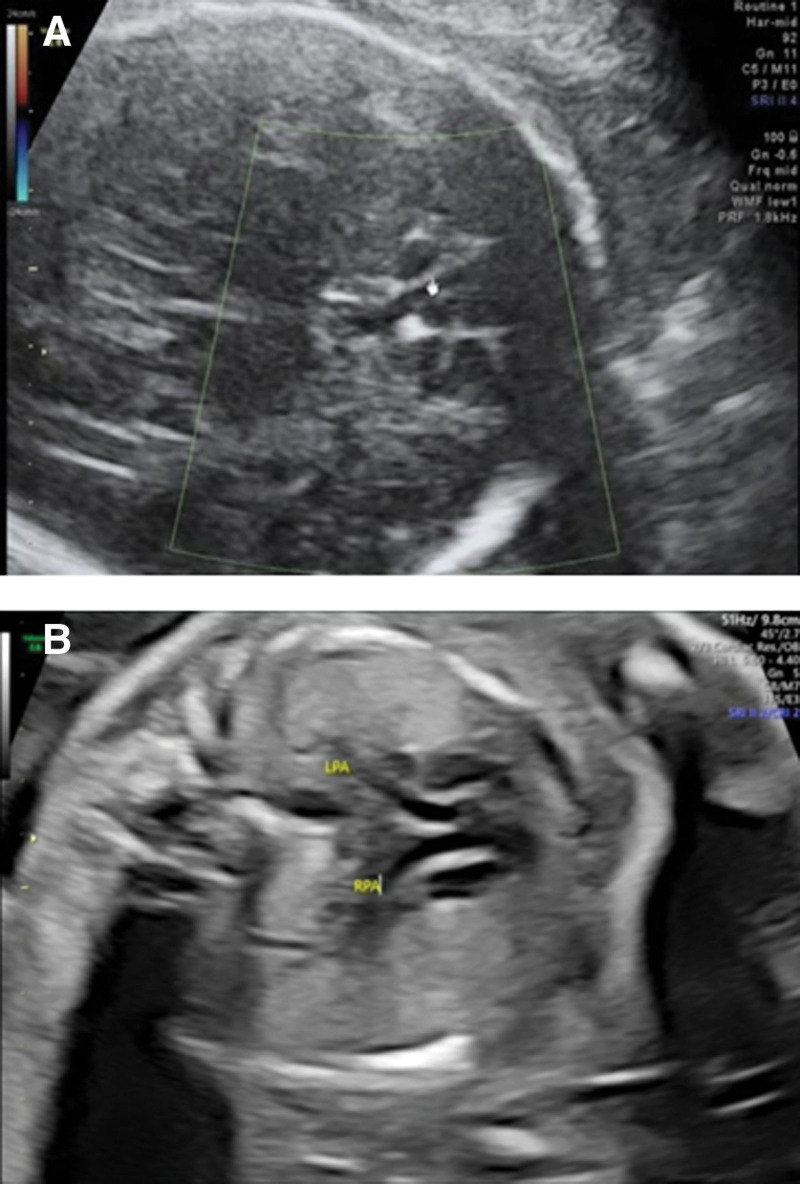
Typical ultrasound performance of the cases. (A) The ultrasound image of Case 1 showed the subependymal cyst. (B) The ultrasound image of Case 2 showed the persistent left superior vena cava.

**Figure 2.**
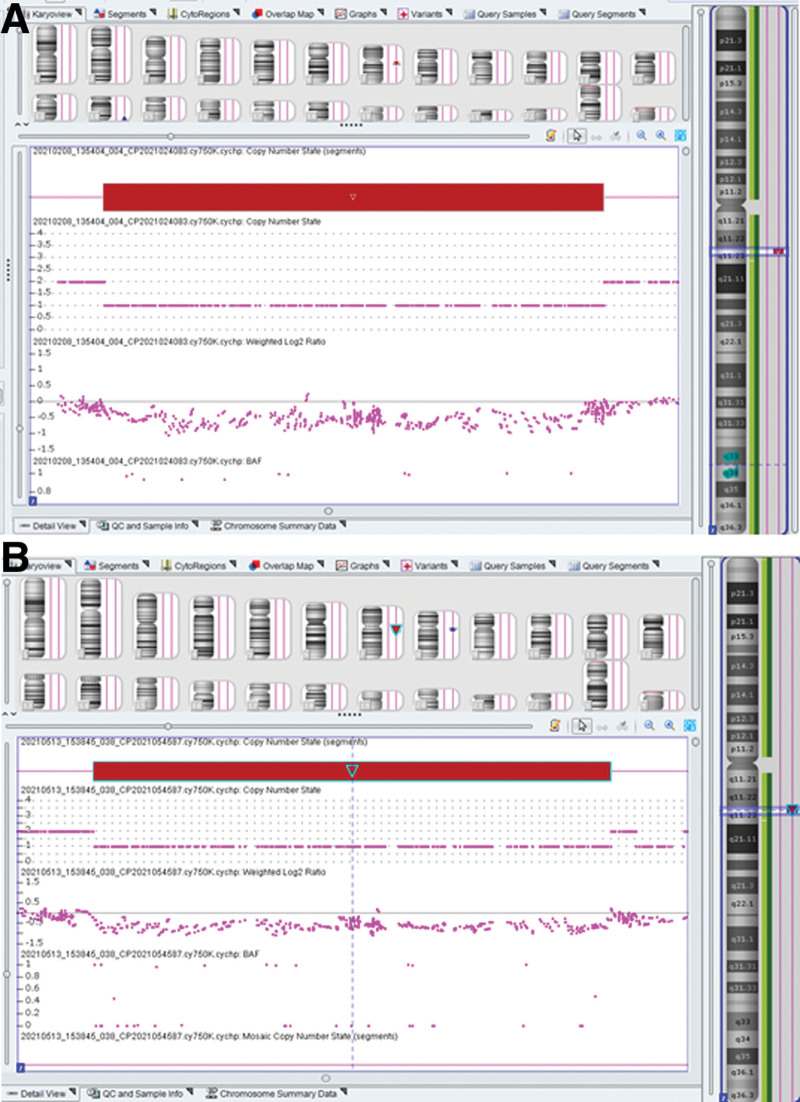
(A) Chromosomal microarray analysis (CMA) array on uncultured amniocytes depicted 7q11.23 (72,677,220–74,213,140) microdeletion. (B) Chromosomal microarray analysis (CMA) array on uncultured amniocytes depicted 7q11.23 (72,745,325–74,154,404) microdeletion.

A 32-year-old woman, gravida 2 para 1 abortion 0, underwent prenatal ultrasound at 25 weeks’ gestation, which presented subependymal cyst (Fig. 1A) in the fetus. The couple are non-consanguineous and healthy. After genetic counseling, the woman underwent amniocentesis at 28 weeks gestation for karyotype analysis and CMA. The karyotype of the fetus was normal. The CMA detected a 1.536Mb deletion in the region of 7q11.23 (arr[hg19] 7q11.23 (72,677,220–74,213,140) × 1) (Fig. 2A). The couple declined karyotyping and CMA analysis verification to confirm the origin of the deletion. Finally, the couple opted for termination of the pregnancy at 32 weeks of gestation according to the genetic counseling.

### 3.2. Case 2

A 31-year-old woman, gravida 2 para 0 abortion 1, underwent prenatal ultrasound at 26 weeks’ gestation, which presented persistent intrauterine growth restriction, persistent left superior vena cava (Fig. 1B) and pericardial effusion (3.4 mm). After genetic counseling, the woman underwent amniocentesis at 27 weeks gestation for karyotype analysis and CMA. The karyotype of the fetus was normal. The CMA detected a 1.409 Mb deletion in the region of 7q11.23 (arr[hg19]7q11..23 (72,745,325–74,154,404) × 1) (Fig. 2B). The couple karyotype analysis and CMA results were normal. The 7q11.23 microdeletion of the fetus was de novo. Finally, the couple opted for termination of the pregnancy at 29 weeks of gestation according to the genetic counseling.

## 4. Discussion

We herein reported 2 prenatal cases with 7q11.23 microdeletion with abnormal ultrasound findings. Case 2 presented intrauterine growth restriction and cardiovascular anomalies, which catered for common prenatal phenotypes of 7q11.23 microdeletions, while case 1 presented subependymal cyst, which was not the atypical phenotypes of 7q11.23 microdeletions. To our knowledge, prenatal phenotype of 7q11.23 microdeletion with subependymal cyst had not been reported before.

7q11.23 microdeletion leads to Williams-Beuren syndrome which was associated with recognizable facial characteristics, intellectual disability, a characteristic friendly personality, and cardiovascular disease.^[16]^ Most reported cases of Williams-Beuren syndrome were adults and children. So far, the prenatal phenotype of Williams-Beuren syndrome is incomplete and atypical.^[17]^

**Figure 3.**
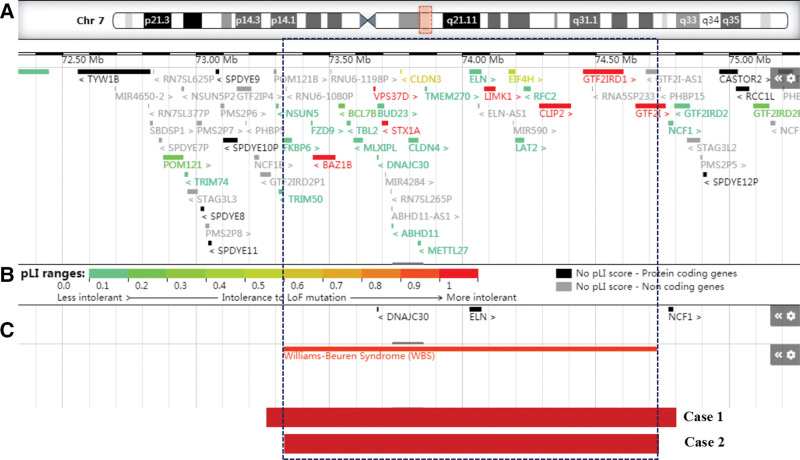
Scale representation of Williams-Beuren syndrome and our cases in the long arm of chromosome 7q11.23 (https://decipher.sanger.ac.uk/). (A) Genes involved in the Williams-Beuren syndrome locus; (B) morbid genes involved in the Williams-Beuren syndrome locus; (C) Deleted fragments in Williams-Beuren syndrome and our cases.

The deleted regions of the 2 fetuses in this study overlapped with the region of Williams-Beuren syndrome (Fig. 3). To better characterize the prenatal genotype–phenotype correlations of Williams-Beuren syndrome, we summarized clinic features of published cases involving pure prenatally detected 7q11.23 deletion (Table 1).^[17–23]^ All 7q11.23 microdeletions varied in size, ranging from 1.06 Mb to 1.98 Mb. Among these cases, 24 cases (No. 9–32) presented apparently normal karyotypes. As summarized, 15/32 cases were de novo and 17/32 cases were not available. Among them, abnormal ultrasound findings were observed in 31/32 cases except no. 10 (high risk of Down syndrome). According to the literature review, the high frequencies of prenatal features were as follows: cardiovascular anomalies (15/32), intrauterine growth restriction (13/32), fetal polycystic kidney (3/32) disease, nuchal-fold thickening (3/32), polyhydramnios (2/32) and pericardial effusion (2/32). Prenatal cardiovascular anomalies in fetal Williams-Beuren syndrome were diverse, including ventricular septal defect (No. 5, 8, 22, 25), tricuspid regurgitation (No. 13, 20), persistent left superior vena cava (No. 26, 32) and pericardial effusion (No. 19, 32). Among these cases, 29/32 cases chose to terminate their pregnancies. 1/32 cases chose to selective fetocide in twin pregnancy, 1/32 cases chose to delivery by cesarean section and 1/32 cases was lost to follow-up. Overall, 7q11.23 microdeletion could manifest various prenatal phenotypes, and it was hard to establish a distinct genotype–phenotype correlation.

**Table 1:** Summary of prenatal features of published cases involving pure 7q11.23 deletion detected by CMA.

No.	Maternal age (yr)	Gestational weeks	Indications for prenatal diagnosis	Karyotype	Chromosome micro array results (hg19)	Deletion size (Mb)	Inheritance	Pregnancy outcome	References
1	31	23	MDCK	n.a.	7q11.23 (72,723,370–74,154,209) × 1	1.43	n.a.	TOP	Huang et al (2022)^[17]^ case 1
2	27	33	AC	n.a.	7q11.23 (72,624,203–74,154,497) × 1	1.53	n.a.	TOP	Huang et al (2022)^[17]^ case 2
3	29	30	DA	n.a.	7q11.23 (72,718,277–74,143,060) × 1	1.42	n.a.	Lost to follow-up	Huang et al (2022)^[17]^ case 3
4	34	22	TOF, SVAS, RAA	n.a.	7q11.23 (72,718,277–74,142,190) × 1	1.42	n.a.	TOP	Huang et al (2022)^[17]^ case 4
5	38	33	VSD, AC	n.a.	7q11.23 (72,718,278–74,143,030) × 1	1.42	n.a.	TOP	Huang et al (2022)^[17]^ case 5
6	33	31	PAS, IUGR	n.a.	7q11.23 (72,557,180–74,628,840) × 1	2.07	n.a.	TOP	Huang et al (2022)^[17]^ case 6
7	23	27	IUGR	n.a.	7q11.23 (72,701,099–74,136,633) × 1	1.44	n.a.	TOP	Huang et al (2022)^[17]^ case 7
8	32	24	VSD	n.a.	7q11.23 (72,723,371–74,141,494) × 1	1.42	n.a.	TOP	Huang et al (2022)^[17]^ case 8
9	24	31	Polyhydramnios	46, XX	7q11.23 (73,028,758–74,090,648) × 1	1.06	n.a.	TOP	Li et al (2021)^[18]^ case 10
10	24	22	High risk of Down syndrome	46, XX	7q11.23 (72,655,376–74,154,209) × 1	1.50	n.a.	TOP	Li et al (2021)^[18]^ case 11
11	30	21	NF thickening	46, XY	7q11.23 (72,765,457–74,257,046) × 1	1.49	n.a.	TOP	Li et al (2021)^[18]^ case 12
12	37	24	MDCK	46, XX	7q11.23 (72,723,370–74,146,927) × 1	1.42	n.a.	TOP	Li et al (2021)^[18]^ case 13
13	31	33	TR	46, XY	7q11.23 (72,713,282–74,154,209) × 1	1.44	n.a.	TOP	Li et al (2021)^[18]^ case 14
14	23	30	NLDC	46, XX	7q11.23 (72,718,123–74,154,209) × 1	1.44	n.a.	TOP	Li et al (2021)^[18]^ case 15
15	27	32	IUGR	46, XN	7q11.23 (72,723,370–74,143,240) × 1	1.42	de novo	TOP	Liang et al (2021)^[19]^ case 1
16	31	26	IUGR, NF thickening, PAS	46, XN	7q11.23 (72,713,282–74,154,209) × 1	1.44	n.a.	TOP	Liang et al (2021)^[19]^ case 2
17	28	30	IUGR	46, XN	7q11.23 (72,650,120–74,154,209) × 1	1.50	de novo	TOP	Liang et al (2021)^[19]^ case 3
18	31	22	Neural tube defect high risk, NF thickening	46, XN	7q11.23 (72,765,457–74,257,046) × 1	1.49	de novo	TOP	Dang et al (2020)^[20]^ case 2
19	24	22	CV widened, strong light echo of left, VTC, PE, high risk of Down syndrome	46, XN	7q11.23 (72,655,376–74,154,209) × 1	1.50	de novo	TOP	Dang et al (2020)^[20]^ case 3
20	31	33	RVS, TR	46, XN	7q11.23 (72,713,282–74,154,209) × 1	1.44	de novo	TOP	Dang et al (2020)^[20]^ case 4
21	37	24	MDCK	46, XN	7q11.23 (72,723,370–74,146,927) × 1	1.40	de novo	TOP	Dang et al (2020)^[20]^ case 5
22	35	23	IUGR, VSD	46, XY	7q11.23 (72,745,047–74,138,460) × 1	1.39	de novo	TOP	Yuan et al (2020)^[21]^ case 1
23	27	22	IUGR, SUA, ICEF in the left ventricle	46, XX	7q11.23 (72,732,834–74,136,633) × 1	1.40	de novo	Delivery by CS	Yuan et al (2020)^[21]^ case 2
24	37	20	IUGR	46, XY	7q11.23 (72,725,759–74,154,209) × 1	1.43	de novo	TOP	Yuan et al (2020)^[21]^ case 3
25	34	23	IUGR, VSD	46, XX	7q11.23 (72,624,166–74,207,565) × 1	1.58	de novo	TOP	Yuan et al (2020)^[21]^ case 4
26	33	24	IUGR, AC, PLSVC	46, XY	7q11.23 (72,765,457–74,175,640) × 1	1.41	de novo	DCDA, selective fetocide	Yuan et al (2020)^[21]^ case 5
27	32	32	IUGR	46, XX	7q11.23 (72,621,722–74,209,949) × 1	1.59	de novo	TOP	Yuan et al (2020)^[21]^ case 6
28	28	24	IUGR, RAA	46, XY	7q11.23 (72,650,120–74,154,527) × 1	1.50	de novo	TOP	Yuan et al (2020)^[21]^ case 7
29	24	31	Polyhydramnios	46, XX	7q11.23 (73,028,758–74,090,648) × 1	1.06	n.a.	TOP	Li et al (2019)^[22]^ case 7
30	30	13	IUGR, omphalocele	46, XY	7q11.23 (72,533,338–74,521,575) × 1	1.98	de novo	TOP	Marcato et al (2014)^[23]^ case 3
31	32	25	Subpendymal cyst	46, XN	7q11.23 (72,677,220–74,213,140) × 1	1.54	n.a.	TOP	Our case1
32	31	26	IUGR, PLSVC, PE	46, XN	7q11.23 (72,745,325–74,154,404) × 1	1.41	de novo	TOP	Our case2

AC = aortic coarctation, CS = cesarean section, CV = Coronary veins, DA = duodenal atresia, DCDA = dichorionic diamniotic, ICEF = intracardiac echogenic focus, IUGR = intrauterine growth restriction, MCDK = multicystic dysplastic kidney, n.a. = not available, NF = nuchal-fold, NLDC = nasal lacrimal duct cyst, PAS = pulmonary artery stenosis, PE = pericardial effusion, PLSVC = persistent left superior vena cava, RAA = right aortic arch, RVS = right ventricle slant, SUA = single umbilical artery, SVAS = supravalvular aortic stenosis, TOF = Tetralogy of Fallot, TOP = termination of pregnancy, TR = tricuspid regurgitation, VSD = ventricular septal defect, VTC = ventricular tendinous cord.

Williams-Beuren syndrome is caused by the pathological loss of the Williams-Beuren syndrome critical region, a 1.55 to 1.83 Mb region that encompasses 25 to 27 unique protein-coding genes on chromosome 7q11.23.^[8]^ According to the DECIPHER database, a total of 10 morbid genes with clinical diseases exist in the region of 7q11.23 (Table 2).

**Table 2:** Morbid genes in the region of 7q11.23 and the associated diseases.

Morbid genes	OMIM	Description	Diseases
ELN	130160	Elastin	Cutis laxa, Supravalvar aortic stenosis
FKBP6	604839	FK506-binding protein 6	Spermatogenic failure 77
DNAJC30	618202	DNAJ/HSP40 homolog, subfamily C, member 30	Leber hereditary optic neuropathy
NCF1	608512	Neutrophil cytosolic factor 1	Chronic granulomatous disease 1
POR	124015	Cytochrome P450 oxidoreductase	Antley-Bixler syndrome with genital anomalies and disordered steroidogenesis, Disordered steroidogenesis due to cytochrome P450 oxidoreductase
MDH2	154100	Malate dehydrogenase, mitochondrial	Developmental and epileptic encephalopathy 51
HSPB1	602195	Heat-shock 27-kD protein 1	Charcot-Marie-Tooth disease (axonal, type 2F), Neuronopathy (distal hereditary motor, type IIB)
YWHAG	605356	Tyrosine 3-monooxygenase/tryptophan 5-monooxygenase activation protein, gamma isoform	Developmental and epileptic encephalopathy 56
ZP3	182889	Zona pellucida glycoprotein 3	Oocyte maturation defect 3
PTPN12	600079	Protein-tyrosine phosphatase, nonreceptor-type, 12	Colon cancer

Among these morbid genes, ELN (OMIM: 130160) gene is the single most important gene that responsible for the cardiovascular anomalies of Williams-Beuren syndrome.^[24]^ Rare alteration of ELN gene produces disease by impacting protein dosage in mechanism of Williams-Beuren syndrome.^[25]^ Elastin insufficiency leads to impaired elastin assembly and inelasticity of the arterial tree, causing increased arterial stiffness and even increased blood pressure.^[26]^ Given that the cardiovascular structure and function is very sensitive to elastin dose, fetuses with Williams-Beuren syndrome may present with varying degrees of cardiovascular abnormalities.^[27]^ The common cardiovascular abnormalities in individuals with Williams-Beuren syndrome is valvar aortic stenosis.^[28]^ In our report, none of the 2 cases presented with typical valvar aortic stenosis, but case 2 presented with other cardiovascular anomalies, including persistent left superior vena cava and pericardial effusion. Huang et al^[17]^ also found that the incidence of supravalvular aortic stenosis were lower than in previous reports. This may be related to the limitations of prenatal ultrasonography.

NCF1 (OMIM: 608512) gene consists of 11 exons that extend over 8 kb and resides at the telomeric end of the Williams-Beuren syndrome critical region.^[8,29]^ NCF1 is a component of the NAD(P)H oxidase complex and is involved in the generation of oxidative stress.^[30]^ Increasing oxidative stress may play a pathophysiological role in cardiovascular disease (hypertension, atherosclerosis, and heart failure) and abnormalities (mainly aortic stenosis).^[31]^ Loss of NCF1 has been associated with relative protection from hypertension and vascular stiffness in individuals with Williams-Beuren syndrome.^[30,32]^ Approximately half of Williams-Beuren syndrome cases develop hypertension.^[33]^ Cases with Williams-Beuren syndrome were observed stiff conducting vessels, even in the youngest children.^[30]^

DNAJC30 (OMIM: 618202) gene has no intron, and it encodes a member of the DNAJ molecular chaperone homology domain-containing protein family. Tebbenkamp et al^[34]^ found that DNAJC30 was enriched in developing and mature neurons where it interacted with the mitochondrial ATP synthase machinery and facilitates ATP synthesis, and linked mitochondria to brain development. Bialleleic missense variation in DNAJC30 is associated with Leber hereditary optic neuropathy in humans.^[35]^ Leber hereditary optic neuropathy is a mitochondrial condition. This mitochondrial condition had not been reported in Williams-Beuren syndrome. But there was an evidence suggesting that decreased DNAJC30 in mice resulted in some phenotypes of Williams-Beuren syndrome, such as thinner callosal axons, social aberrations, and increased anxiety.^[34]^

In addition, 7q11.23 deletion (Williams-Beuren syndrome) is one of the most frequently pathogenic CNVs of fetuses with growth restriction, particularly in isolated FGR.^[36]^ Intrauterine growth retardation was also found in our case 2. Yuan et al^[21]^ reported that 82.35% (14/17) cases of prenatal Williams-Beuren syndrome combined with intrauterine growth retardation. However, in our report, the incidence of intrauterine growth retardation in prenatal Williams-Beuren syndrome was 40.63% (13/32) based on our literature review. Williams-Beuren syndrome presented specific growth pattern, characterized by intrauterine growth restriction, low weight, length, and head circumference at birth, and this global growth delay persisted during childhood and adolescence.^[37]^

Of note, subependymal cyst was found in our case 1. To our knowledge, this manifestation was first-reported as an ultrasound manifestation of Williams-Beuren syndrome. There are 2 types of subependymal cysts, one of which is an acquired, posthemorrhagic cyst and the other of which is congenital and is related to germinolysis.^[38]^ Although isolated subependymal cyst are usually a benign finding, further investigations is required.^[39]^

In this study, we reported 2 prenatal cases with 7q11.23 microdeletion (Williams-Beuren syndrome), in which the fetal subependymal cyst in case 1 has not been mentioned in other reports. This manifestation may expand prenatal phenotype of Williams-Beuren syndrome. Given the phenotypic diversity of Williams-Beuren syndrome, comprehensive interpretation and genetic counseling for 7q11.23 microdeletion remain challenging. Comprehensive interpretation and genetic counseling of 7q11.23 microdeletion should consider the combination of prenatal ultrasound screening and traditional cytogenetic and molecular genetic analysis.

## Acknowledgments

We are grateful to all the participants for consenting to participate in this study.

## Author contributions

**Conceptualization:** Xin Lv, Ruixue Wang.

**Data curation:** Xiao Yang.

**Formal analysis:** Xiao Yang.

**Investigation:** Linlin Li.

**Methodology:** Fagui Yue.

**Supervision:** Hongguo Zhang.

**Validation:** Hongguo Zhang.

**Writing – original draft:** Xin Lv.

**Writing – review & editing:** Ruixue Wang.
